# How AlphaFold2 Predicts Conditionally Folding Regions Annotated in an Intrinsically Disordered Protein Database, IDEAL

**DOI:** 10.3390/biology12020182

**Published:** 2023-01-25

**Authors:** Hiroto Anbo, Koya Sakuma, Satoshi Fukuchi, Motonori Ota

**Affiliations:** 1 Faculty of Engineering, Maebashi Institute of Technology, Maebashi 371-0816, Japan; 2 Graduate School of Informatics, Nagoya University, Nagoya 464-8601, Japan; 3 Institute for Glyco-core Research, Nagoya University, Nagoya 464-8601, Japan

**Keywords:** protein structure prediction, intrinsically disordered regions, database, assessment of prediction

## Abstract

**Simple Summary:**

Intrinsically disordered regions (IDRs) in intrinsically disordered proteins (IDPs) play important roles in various biological processes by providing protein binding regions. The regions can adopt local structures upon binding to their interaction partners. An IDP database—IDEAL—has collected these conditionally binding regions as Protean Segments (ProSs). A recently developed program, called AlphaFold2 (AF2), accurately predicts structural domains in proteins. Because ProSs have the bilateral characteristics of IDRs and ordered regions, assessing AF2 models corresponding to ProSs is worthwhile. We classified ProSs into three classes: the excellent class agrees well with the AF2 models, the poor class agrees poorly, and the average class agrees between these two. The ProSs in the excellent class were characterized by some features similar to globular structures, whereas those in the poor class showed features of extended structures. The ProSs in the excellent class were further grouped into those with high prediction reliability (pLDDT) and those with a relatively low pLDDT and a small normalized radius of gyration.

**Abstract:**

AlphaFold2 (AF2) is a protein structure prediction program which provides accurate models. In addition to predicting structural domains, AF2 assigns intrinsically disordered regions (IDRs) by identifying regions with low prediction reliability (pLDDT). Some regions in IDRs undergo disorder-to-order transition upon binding the interaction partner. Here we assessed model structures of AF2 based on the annotations in IDEAL, in which segments with disorder-to-order transition have been collected as Protean Segments (ProSs). We non-redundantly selected ProSs from IDEAL and classified them based on the root mean square deviation to the corresponding region of AF2 models. Statistical analysis identified 11 structural and sequential features, possibly contributing toward the prediction of ProS structures. These features were categorized into two groups: one that contained pLDDT and the other that contained normalized radius of gyration. The typical ProS structures in the former group comprise a long α helix or a whole or part of the structural domain and those in the latter group comprise a short α helix with terminal loops.

## 1. Introduction

Proteins play essential biological roles in all organisms. Because knowledge of protein three-dimensional (3D) structures can facilitate understanding of their molecular functions [1], almost 200,000 protein structures and structural complexes have been solved and deposited in the Protein Data Bank (PDB) [2]. Because experimental procedures to determine protein structures are laborious and time consuming, methods to predict protein structure have been studied for half a century [3]. The structural knowledge of all proteins would accelerate biological studies tremendously. AlphaFold2 (AF2) [4] is a protein structure prediction method based on state-of-the-art techniques of machine learning. The outstanding performance of AF2 was validated in the 14th Critical Assessment of techniques for protein Structure Prediction (CASP14) [3]. AF2 is freely available as an open resource and can be installed on our local platforms. It was applied to proteomes of some model organisms (AlphaFold Protein Structure Database, AlphaFold DB) [5] and the representative UniProt sequences [6]; the models of these proteins are available to the public. Through these services, one can access model structures by AF2 (AF2 models) for almost all known proteins.

AF2 provides accurate protein 3D structures for foldable proteins. However, intrinsically disordered proteins (IDPs) contain intrinsically disordered regions (IDRs) that do not adopt 3D structures [7,8,9,10]. IDPs are known to play significant cellular roles, especially signal transduction and transcription [7,8,11]. They are abundant in eukaryotic proteins, particularly nuclear proteins [12,13]. Some IDPs are fully disordered and contain only one IDR from the N- to C-terminus. Other IDPs comprise a mixture of IDRs and structural domains. Some long IDRs contain functional regions that conditionally fold into specific structures upon binding to interaction partners. This disorder-to-order transition is characteristic of IDPs and is known as the coupled folding and binding mechanism [8]. The conditionally folding regions are called short linear motif (SLiM) [14], molecular recognition feature (MoRF) [15], or disordered binding sites (DIBS) [16]. Over the past decade, we have been constructing and managing an IDP database called IDEAL (https://www.ideal-db.org, accessed on 20 January 2023) [17,18]. In IDEAL, disordered regions are judged by reading original manuscripts, and they essentially correspond to missing residues in the X-ray structures, or regions that have been shown to be flexible in experiments using NMR, CD, and other methods. IDEAL collects such experimentally verified IDRs and structural domains in IDPs, as well as conditionally folding regions. We call these regions Protean Segments (ProSs).

As mentioned above, IDPs are abundant in eukaryotic proteins. AF2 has been applied to them and monomeric AF2 models are provided in AlphaFold DB [5]. Thus, IDPs, including ProSs, should be found in the AF2 models. We defined ProS in IDEAL as when disordered and ordered experimental evidences are available for a single region. The ordered experimental evidence is usually the complex structure of the ProS and its partner [17]. Most such structures were deposited in the PDB before the learning process of AF2. Keeping these situations in mind, how AF2 predicts the regions for ProSs in their monomeric models is unclear. Because AF2 has learned structures in the PDB, it may predict ProS structures in complex forms, even though AF2 models themselves are in monomeric forms. By contrast, it is unclear whether AF2 successfully builds models for all ProSs, because ProSs have different structures and sequences. Knowing the inclination of AF2 models for ProSs would facilitate the identification of unknown ProSs, e.g., locations in the sequences and interaction partners. In this study, we constructed non-redundant ProS datasets from IDEAL and investigated how they were predicted by AF2. We compared experimental ProS structures with the corresponding regions in the AF2 models, and classified ProSs using root mean square deviations (RMSDs) between them. We found that one-third of AF2 models agreed well with ProS structures, whereas one-third did not. We examined structural and sequential features of ProSs that agreed well and found two groups: one is characterized by high prediction reliability (predicted local distance difference test: pLDDT) and another by relatively low pLDDT and a small normalized radius of gyration (nRG).

## 2. Materials and Methods

### 2.1. Dataset

We constructed a dataset of non-redundant ProSs from proteins in IDEAL, in which 346 intrinsically disordered proteins contained ProS(s). From these proteins, we selected eukaryotic proteins for which AF2 models were provided by AlphaFold DB. ProSs were labeled as verified, possible, or predicted in IDEAL according to their reliability [17], and we only used the first two types. ProSs which were at most 10 residues long were discarded. When some ProSs were redundantly assigned around a region in a protein, the longest one was selected. We confirmed interactions of ProSs with their interaction partner(s) through accessible surface area (ASA) calculated by DSSP [19]. ASAs were calculated for a ProS with and without interaction partner(s). Their difference was defined as ΔASA. We discarded complex structures of ProS if ΔASA was 0. The full lengths of protein sequences including selected ProSs were clustered using BlastClust [20] with 30% sequence identity and 90% overlap, and the proteins with the longest ProSs was selected. Finally, 164 proteins containing 207 ProSs and their interaction partners were selected (Table S1).

### 2.2. Classification of ProS Structures

We compared experimental ProS structures and AF2 models and calculated the RMSD of Cα atoms between them. According to the RMSD values, ProSs were divided into three classes: excellent (small RMSD (≤1.76 Å)), average, and poor (large RMSD (≥4.13 Å)). The average class was assumed to be an allowance to emphasize the differences between the excellent and poor classes. TM-score [21] is a sequence-length-independent measure of structural similarity; however, there are short ProSs whose sequence length is 11 at most. Because TM-scores for these short ProSs cannot be defined, we could not use the TM-score in this study. The boundaries of RMSDs between the excellent, average, and poor classes were decided so that the structural and sequential features (see below) of each class were most distinctive using the *p*-value of the Mann–Whitney *U*-test (Appendix A). This test compares distributions of two classes and evaluates whether two distributions are similar or not. A significantly small *p*-value (< 0.01) means that the distributions of a feature value in two classes are different. Notably, the structures of ProSs were determined with partner proteins, whereas the AF2 models were predicted in the monomeric form.

### 2.3. Structural and Sequential Features

We considered 38 structural and sequential features in total (Table S2). The features included amino acid composition, rates of secondary structures (α, β, coil), and relative ASA (rASA) of an experimental structure of ProS, the number of homologous sequences, and averaged pLDDT over a ProS region in the AF2 model, etc. We evaluated which features efficiently differentiated ProSs in the excellent class. The distributions of a feature in the excellent and poor classes were compared, and if the *p*-value of the Mann–Whitney *U*-test was significantly small (0.01), the feature was considered efficient.

### 2.4. Characterization of ProSs in the Excellent Class

Using efficient structural and sequential features as explanatory variables, we conducted multiple regression analyses to infer RMSDs between experimental ProS structures and corresponding regions of AF2 models. We estimated the contributions of each feature on the regression.

To evaluate the relationships between the features, we calculated the correlation coefficients of feature pairs and constructed a dendrogram using the Ward method. In the dendrogram, the values of 1 minus the correlation coefficients (from 0 to 2) were used as the distance. Some features showed negative correlations to RMSDs, and others, positive. To remove the effect of anti-correlation between features, all features were arranged to increase along the RMSD. Specifically, we took minus values of features if the feature showed negative correlation with RMSDs.

### 2.5. Software

The statistical tests were performed using the SciPy library [22] of the Python 3 language [23]. All of the violin plots, a dendrogram, and all of the figures for molecular structures were created by the Seaborn library [24] of the Python 3 language, R language [25], and PyMOL [26], respectively.

## 3. Results and Discussions

### 3.1. ProSs Agreeing with AF2 Models

#### 3.1.1. Structural and Sequential Features to Differentiate between Excellent and Poor Classes

**Figure 1:**
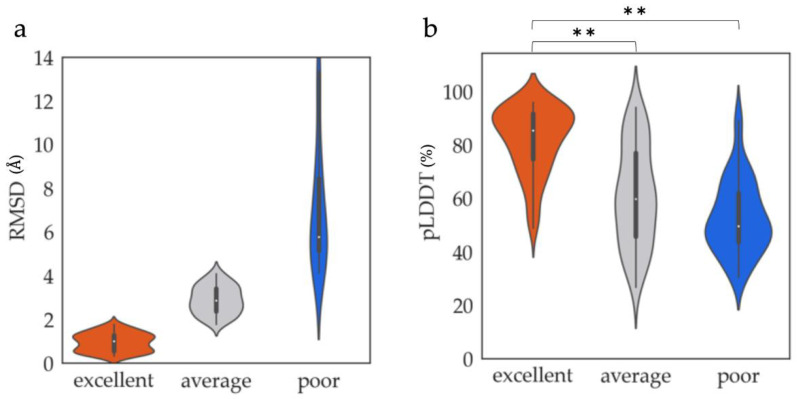
Comparisons of the ProS classes. (**a**) Distributions of RMSDs between the experimental structures of ProSs and the corresponding regions in AF2 models for the excellent, average, and poor classes. The excellent and average classes and average and poor classes were divided by RMSDs of 1.76 and 4.13 Å, respectively. (**b**) Distributions of averaged pLDDT over the ProS regions in AF2 models for the excellent, average, and poor classes. Double asterisks (**) represent a significant difference between a pair of distributions with a *p*-value less than 0.01, with the Mann–Whitney *U*-test.

The classification results revealed that 63, 82, and 62 ProSs were categorized into excellent, average, and poor classes, respectively. The distribution of RMSDs for each class is shown in Figure 1a. The RMSDs in the excellent class were distributed in a range of small RMSDs, indicating that AF2 can predict one-third of the ProS structures binding to the partner proteins even though AF2 models were provided in monomeric forms. By contrast, another one-third of the ProSs were in the poor class, which means that they did not agree with the AF2 models. The results indicated that there are ProSs (the excellent class) that can be accurately predicted by AF2.

Next, we examined the types of ProSs that were predicted by AF2. We considered 38 structural and sequential features (Table S2; see Methods) as candidates to characterize ProSs in the excellent class. The distribution of a given feature was obtained for the excellent, average, and poor classes, and the differences of the distributions were evaluated using the *p*-value of the Mann–Whitney *U*-test. Figure 1b shows an example of a feature, the averaged-pLDDT, which represents average values of pLDDTs over residues comprising a ProS. In this case, the excellent class contained many ProSs whose averaged pLDDTs were high. By contrast, ProSs with low averaged pLDDTs were abundant in the poor class. In the average class, the values were widely distributed. The distributions of the excellent and poor classes, as well as the excellent and average classes, were significantly different (less than 0.01 *p*-value). Similar to this example, we evaluated the distributions of 38 features. The results of the Mann–Whitney *U*-test are summarized in Table S2. Finally, we selected 11 features, for which the *p*-values between the excellent and poor classes were significantly small (see violin plots in Figure S1). They were averaged pLDDT (hereafter, pLDDT), rASA in the monomeric (without interaction partner) and complex (with interaction partner) forms (mrASA and crASA, respectively), nRG (Appendix B), fractions of residues in α helices and coil regions (%Helix and %Coil, respectively), fractions of polar (G, N, P, Q, S, T) and hydrophobic (A, C, I, L, M, V) amino acid residues (hydrophobic and polar, respectively), and fractions of residues of A, L, and S. Notably, except for the pLDDT of AF2 models, the experimental ProS structures were employed to calculate the structural features.

#### 3.1.2. Two Types of ProSs in the Excellent Class

**Figure 2:**
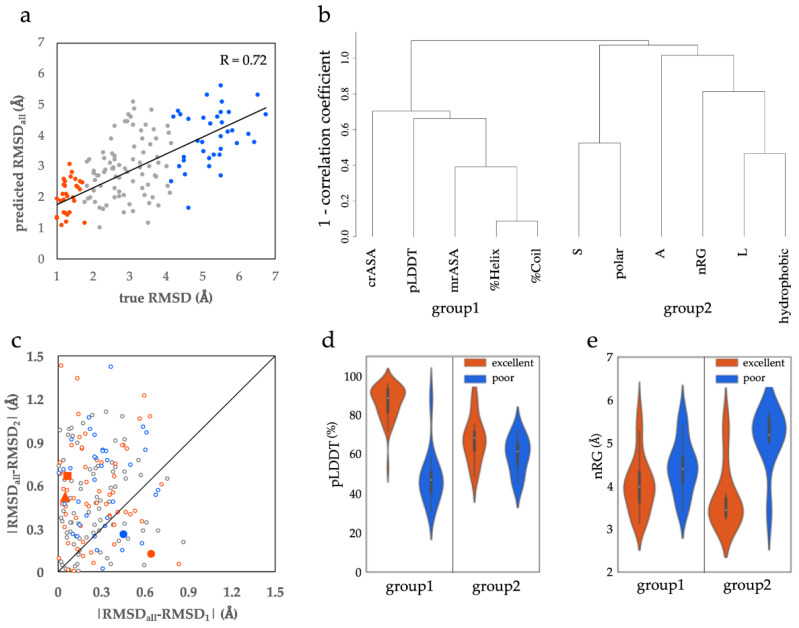
Grouping ProSs by 11 efficient features. (**a**) The predicted RMSDs, inferred using multiple regression with 11 features, were plotted against true RMSDs. The outliers were excluded in the multiple regression (see Figure S2). Blue, gray, and red dots represent ProSs in excellent, average, and poor classes, respectively. (**b**) Clustering of 11 features. Features located on the left and right sides are in groups 1 and 2, respectively. (**c**) Scatter plots of the absolute differences of regression results using all features and those using group 1 or 2 features. The diagonal line divides the ProSs into group 1 (upper) and group 2 (lower). Dots are colored in the same manner in the panel a). Large red and blue symbols indicate the values of examples for the ProSs in the excellent and poor groups, respectively, described in 3.1.4 and 3.1.5 (red triangle (PDB_ID: 1pq1B), red square (3i91A), red circle (2czyB), and blue circle (4rjfB)). (**d**) Distributions of pLDDT of group 1 and group 2 ProSs in the excellent and poor classes. (**e**) Distributions of nRG of group 1 and group 2 ProSs in the excellent and poor classes.

We identified 11 features that could discriminate the ProSs in the excellent class from the ones in the poor class. Because the ProS classes were defined based on RMSD values between ProS structures and the corresponding AF2 models, we could infer the RMSDs by using the 11 features. We conducted multiple regression to deduce the RMSDs using the 11 features as explanatory variables and evaluated the contributions of each feature to the inference. We obtained a regression model with 0.31 *R*-squared and 0.55 correlation coefficients (Figure S2). These small values simply indicated that a linear fitting of the RMSDs would be difficult. However, we noticed outliers in the region with more than 7 Å RMSD (Figure S2). When these outliers were ignored and multiple regression was conducted again, the model showed 0.52 *R*-squared and 0.72 correlation coefficient (Figure 2a). The coefficients and t-values of this model are shown in Table 1. Because the absolute ranges of the features differed, e.g., pLDDT from 0 to 100 and %Coil from 0 to 1, the coefficients themselves could not be directly compared. By contrast, the t-values represented the contributions of the variables to the regression, and their absolute values represented the significance. pLDDT contributed the most to the regression of the RMSDs, followed by nRG.

In the process of the regression, we found that some features correlated with each other. To evaluate the relationship between the 11 features, we conducted cluster analysis using the Ward method (Figure 2b). The dendrogram showed that the features were divided into two groups. crASA, pLDDT, mrASA, %Helix, and %Coil constituted group 1 (the left part of Figure 2b), and the fractions of S, polar, A, L, and hydrophobic, and nRG comprised group 2 (the right part). pLDDT in group 1 and nRG in group 2 were the features contributing the most for the multiple regression in each group (Table 1). These results suggest the presence of two types of ProSs, each of which is characterized by the features in group 1 or 2. Then, we tried to identify ProSs characterized by the features of groups 1 and 2.

When some features were significant to characterize a given ProS, omitting those features was expected to make the regression results worse. By contrast, the regression result would not be largely altered by omitting the features poorly characterizing that ProS. We inferred the RMSDs by using the same regression model again, but in two ways. For the first inference, we only used the features in group 1 and converted the coefficients for the features in group 2 into zero to omit their effects. The RMSDs obtained by this procedure were denoted by RMSD_1_. The second inference was performed with only the features in group 2 (RMSD_2_). We compared these inferred RMSDs with those inferred by using all features (RMSD_all_), namely the RMSDs obtained by the original regression model. Figure 2c shows the comparison, where the differences of inferred RMSDs, |RMSD_all_–RMSD_1_| and |RMSD_all_–RMSD_2_| were plotted. We divided all ProSs into two types with the diagonal line. The ProSs plotted in the upper diagonal region were more affected by omitting the features in group 1 and were named group 1 ProSs. The others were affected by the features in group 2 and were named group 2 ProSs.

The distributions of pLDDT and nRG of group 1 and 2 ProSs are shown in Figure 2d and 2e, respectively. In group 1 ProSs, pLDDT clearly discriminated the excellent class from the poor class (Figure 2d), whereas it did not in group 2 ProSs. Conversely, nRG differentiated the excellent and poor classes well only in group 2 ProSs (Figure 2e). The results suggested that in group 1 ProSs, the excellent class can be discriminated from the poor class principally by pLDDT, and in group 2, nRG discriminated the excellent class from the poor one. This indicates that the ProS groups defined in Figure 2c reflected the groups of features well (Figure 2b).

The features in group 1 were characterized by high pLDDT and small mrASA (Figure 2d and Figure S3B), whereas the ones in group 2 were characterized by small nRG (Figure 2e). %Helix discriminated the excellent and poor classes in both group 1 and 2 ProSs (Figure S3C). The features are characteristic of folded proteins, where α helices are stabilized by the main-chain hydrogen bonds that connect sequentially neighboring residues and a compact shape of protein structure (small nRG) is adopted. The results suggested that the ProSs in the excellent class may have the potential to fold independently, irrespective of the group they belong to. Because AF2 learned huge amounts of folded protein structures, it would be reasonable to assume that the ProSs in the excellent class show structural features similar to those of folded proteins. We also speculate that AF2 generates one of the structures in the conformational ensemble of IDPs with higher existence probability. Intriguingly, group 2 ProSs had relatively low pLDDT values and agreed highly with the AF2 models. Although pLDDT is a measure for the confidence of structural models, the results suggest that AF2 can provide good models for conditionally folding segments, regardless of relatively low pLDDT.

#### 3.1.3. Features of the Poor Class ProSs

ProSs in the poor class mostly showed the opposite trends to those in the excellent class, namely lower pLDDT, larger nRG and rASA, lower α helix fractions, and hydrophilic sequences (Figure S1). These features indicated unfolded structures, in which peptide chains take loopy and extended structures. Because some ProSs bind their binding partners in loopy and extended forms, they may belong to the poor class. It is reasonable to assume that AF2 can predict ProS structures with similar features of folding regions and not those with the opposite nature, because AF2 is designed to predict folded structures.

#### 3.1.4. Examples of ProSs in the Excellent Class

We visually inspected two groups of ProSs in the excellent class and extracted the typical structures of the ProSs. Although general features of group 1 ProSs in the excellent class are described above, we found that they could be further categorized into two structural types. One type comprises proteins with one or more long helices (group1_LH). Helices tend to be predicted with high pLDDT and a rather small mrASA, because the main chains are buried. The other type (group1_SD) contains proteins with whole or partial structural domains, which also have small mrASA and tend to be predicted to have high pLDDT.

**Figure 3:**
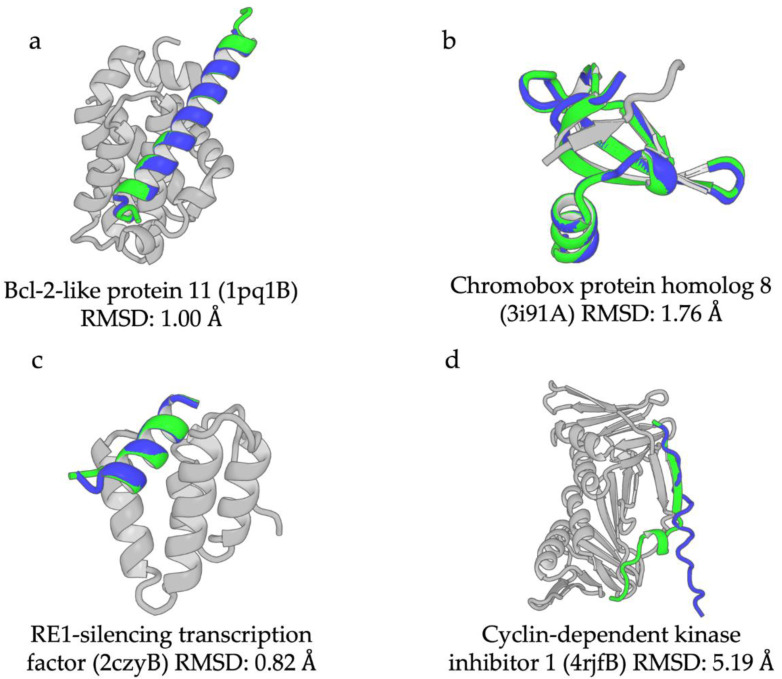
Examples of ProSs in the excellent and poor classes. ProS, the binding partner of ProS, and the AF2 model are represented in green, gray, and blue, respectively. (**a**–**c**) Examples of ex-cellent and (**d**) poor classes. (**a**) The ProS in Bcl-2-like protein 11 (residues 139–171) comprises a long α helix (1pq1B). (**b**) The ProS in chromobox protein homolog 8 (residues 9–60) comprises an entire structural domain (3i91A). (**c**) The ProS in RE1-silencing transcription factor (residues 43–57) comprises a short helix with terminal loops (2czyB). (**d**) The ProS in cyclin-dependent kinase inhibitor 1 (residues 141–160) comprises an extended shape (4rjfB).

A representative example of group1_LH is Bcl-2-like protein 11 (UniProt AC:054918, IDEAL ID: IID50303), commonly called Bim (red triangle in Figure 2c). Bim is a pro-apoptotic protein that interacts with the anti-apoptotic proteins Bcl-Xl of the Bcl-2 family, and acts as an inhibitor of Bcl-Xl [27]. A region from 139–171 in Bim comprises a long helix (in green, PDB ID:1pq1B; Figure 3a), including the BH3 domain, that binds to the groove of Bcl-Xl, which is composed of eight helices (gray, 1pq1A). The region was shown to be disordered in the isolated form using CD [28]. The values for pLDDT, mrASA, and %Helix were 82.2%, 0.47, and 87.1%, respectively. The RMSD between the AF2 model (blue) and the PDB structure was 1.00 Å.

An example of group1_SD is chromobox protein homolog 8 (Q9HC52, IID00682), commonly called Cbx8 (red square in Figure 2c). Cbx8 is one of the eight mammalian Cbx proteins, which are chromodomain-containing proteins involved in the regulation of heterochromatin, gene expression, and developmental programs [29]. The chromodomain (Figure 3b, green, 3i91A) at the N-terminal region of Cbx8 binds with the trimethylated H3K9 (H3K9 me3) peptide (gray, 3i91C). This interaction forms an antiparallel β-sheet between them. The whole chromodomain is a ProS, because it is an IDR in the absence of the peptide [29]. The values for pLDDT and mrASA are 94.9% and 0.389, respectively. The RMSD between AF2 model (blue) and the PDB structure was 1.76 Å.

An example of group 2 ProSs comprises proteins having a short helix with terminal loops. Their nRGs are rather small compared with the long helix found in the ProSs in group1_HL, whereas their pLDDT is not high, probably due to their length. A typical example is RE1-silencing transcription factor (REST) (UniProt AC: Q13127, IDEAL ID: IID00169), which represses the transcription of neuron-specific genes in non-neuronal cells and neuronal progenitors (red circle in Figure 2c). This phenomenon is triggered by the association of the repressor domain (RD-1) of REST in its N-terminal region (Figure 3c, green, 2czyB) with the PAH1 domain of Sin3 (gray, 2czyA) [30,31]. The region of RD-1 is also reported as an IDR [32]. Despite relatively low pLDDT (63.5%), the nRG of the ProS is significantly low (3.25) compared with that of Bim (4.40). The RMSD between the AF2 model (blue) and the PDB structure was 0.82 Å.

#### 3.1.5. Examples of ProSs in the Poor Class

The cyclin-dependent kinase inhibitor 1, p21 (UniProt AC: P38936, IDEAL ID: IID00043) is a typical example of the poor class (blue circle in Figure 2c). This protein plays an important role in regulating cell-cycle progression. Moreover, it inhibits DNA replication by interacting with proliferating cell nuclear antigen (PCNA) [33]. Many proteins, including p21, have a conserved motif called PCNA-interacting protein (PIP) box [34]. The PIP box in the C-terminal of p21 (Figure 3d, green, 4rjfB) binds to the interdomain connector loop (IDCL) linking two similar domains of PCNA (gray, 4rjfA) [33]. This interaction forms an antiparallel β-sheet between p21 and IDCL (Figure 3d). The ProS of p21 partially forms the β-sheet with its partner. This type of interaction is frequently observed in ProSs in the poor group, with higher mrASA and nRG and lower pLDDT and %Helix. The rmASA, nRG, pLDDT, and %Helix were 0.639, 4.99, 65.9%, and 15.0%, respectively. The RMSD between the AF2 model (blue) and the PDB structure was 5.19 Å.

### 3.2. Comparison with Other Assessments of Conditionally Folding Regions

The conditionally folding regions in the AF2 models have been investigated in several studies. Alderson et al. [35] examined about 10 conditional folding regions of AF2 models in the monomeric form, and discussed their plasticity and function. They reported that the structures of some conditional folding regions were similar to those in one of their PDB models, regardless of the relevant experimental conditions. In addition, they found that predicted IDRs contained regions with high pLDDT, and such regions were enriched in the α helix, especially in long helices, and hydrophobic and charged residues. We speculated that these regions corresponded to ProSs in the excellent class (group1_LH), although the charged resides were not significant (Table S2). In the manuscript, the pLDDTs of conditional folding regions are provided. We noticed that the distribution of pLDDTs of ProSs in IDEAL is similar to their result [36,37]. The distribution of pLDDT in this study appears to be bimodal (Figure S4), indicating that two groups schematically corresponded to the excellent and poor groups. Akdel et al. [38] assessed 14 structural complexes of SLiM [14] and the binding partner generated by AF2 and reported that the results agreed remarkably well. We did not evaluate AF2 models of structural complexes in this study; however, the studies are ongoing.

## 5. Conclusions

We assessed AF2 models in the monomeric form for the ProS regions in IDEAL. Some models showed good agreements with the ProS structures and the others did not. The ProSs in the excellent class possessed some features of folded structures such as high pLDDT, small nRG, large fractions of α helix, etc., whereas the ones in the poor class comprised extended structures with low pLDDT and large fractions of coil regions, etc. The ProSs in the excellent class were further characterized by group 1 being dominated principally by pLDDT and group 2 by nRG. This is the first report to systematically assess AF2 models by employing a considerable number of experimentally verified conditionally folding IDRs. The results of this study provide a new aspect of AF2 models.

## Figures and Tables

**Table 1:** Contribution of each feature to the regression model.

	coef	t
pLDDT	0.050	8.979
nRG	0.514	4.143
constant term	5.007	3.614
mrASA	−3.931	−2.997
crASA	2.261	2.081
%Coil	1.273	1.682
L	2.302	1.572
S	1.819	1.387
polar	−0.374	−0.440
hydrophobic	0.516	0.428
%Helix	−0.242	−0.337
A	0.380	0.218

coef, coefficients for each explanatory variable; t, t-values for the t-tests.

## Data Availability

All data generated or analyzed during this study are included in this published article and its supplementary information files.

## Supplementary Materials

The following are available online at https://www.mdpi.com/article/10.3390/biology12020182/s1, Figure S1: Distributions of structural and sequential features of ProS for excellent, average, and poor classes in violin plots, Figure S2: Multiple regression of RMSDs for all ProSs using 11 features, Figure S3: Distributions of structural and sequential features of group 1 and 2 ProSs in the excellent and poor classes, Figure S4: Distribution of pLDDT of non-redundant ProSs in IDEAL, Table S1: List of non-redundant ProSs in IDEAL used in this study, Table S2: Candidates of structural and sequential features characterizing ProSs.

## Appendix A

We classified ProSs into excellent (small RMSD), average, and poor (large RMSD) classes. When groups were well defined, a given feature in one group must differ from those in the other groups. We determined the boundary values of RMSD to divide ProSs so that the distributions of the 38 features were the most different between classes. We conducted the Mann–Whitney *U*-test to compare the distributions of two groups (38 × 3C2 = 114 pairs). The classifications were exhaustively evaluated (20% to 40% population for excellent and poor classes by 2% increment). We searched for the threshold RMSD in which the number of significantly different distributions (less than 0.01 *p*-value after applying Bonferroni’s correction) was the highest, and the best divisions were obtained (1.76 Å for the excellent and average boundary and 4.13 Å for the average and poor boundary).

## Appendix B

Radius of gyration (RG) is a measure of the extendedness (or globularity) of polymer molecules, which corresponds to the radius when the molecule is approximated by a sphere. Because RG depends on the length of the polymer, the comparison of RGs between proteins requires normalization by protein length. We defined normalized RG by $nRG=RG/N^{\frac{2}{5}}$, according to Di Cola et al. [39].
