# Supplementary material for: How AlphaFold2 Predicts Conditionally Folding Regions Annotated in an Intrinsically Disordered Protein Database, IDEAL
Source: Biology (Basel). 2023 Jan 25;12(2):182. doi: 10.3390/biology12020182 (PMC9952413; doi:10.3390/biology12020182)
Supplement: Supplementary file 1 [file biology-12-00182-s001.zip › biology-2153021-supplementary.pdf]

Supplemental Figures

How AlphaFold2 predicts disordered regions and conditionally folding regions annotated in an intrinsically-disordered protein database, IDEAL

Hiroto Anbo, Koya Sakuma, Satoshi Fukuchi, Motonori Ota

Faculty of Engineering, Maebashi Institute of Technology, Maebashi 371-0816, Japan
Graduate School of Informatics, Nagoya University, Nagoya 464-8601, Japan

Figure S1: Distributions of structural and sequential features of ProS for excellent, average, and poor classes in violin plots.
Figure S2: Multiple regression of RMSDs for all ProSs using 11 features.
Figure S3: Distributions of structural and sequential features of group 1 and 2 ProSs in the excellent and poor classes.
Figure S4: Distribution of pLDDT of non-redundant ProSs in IDEAL.

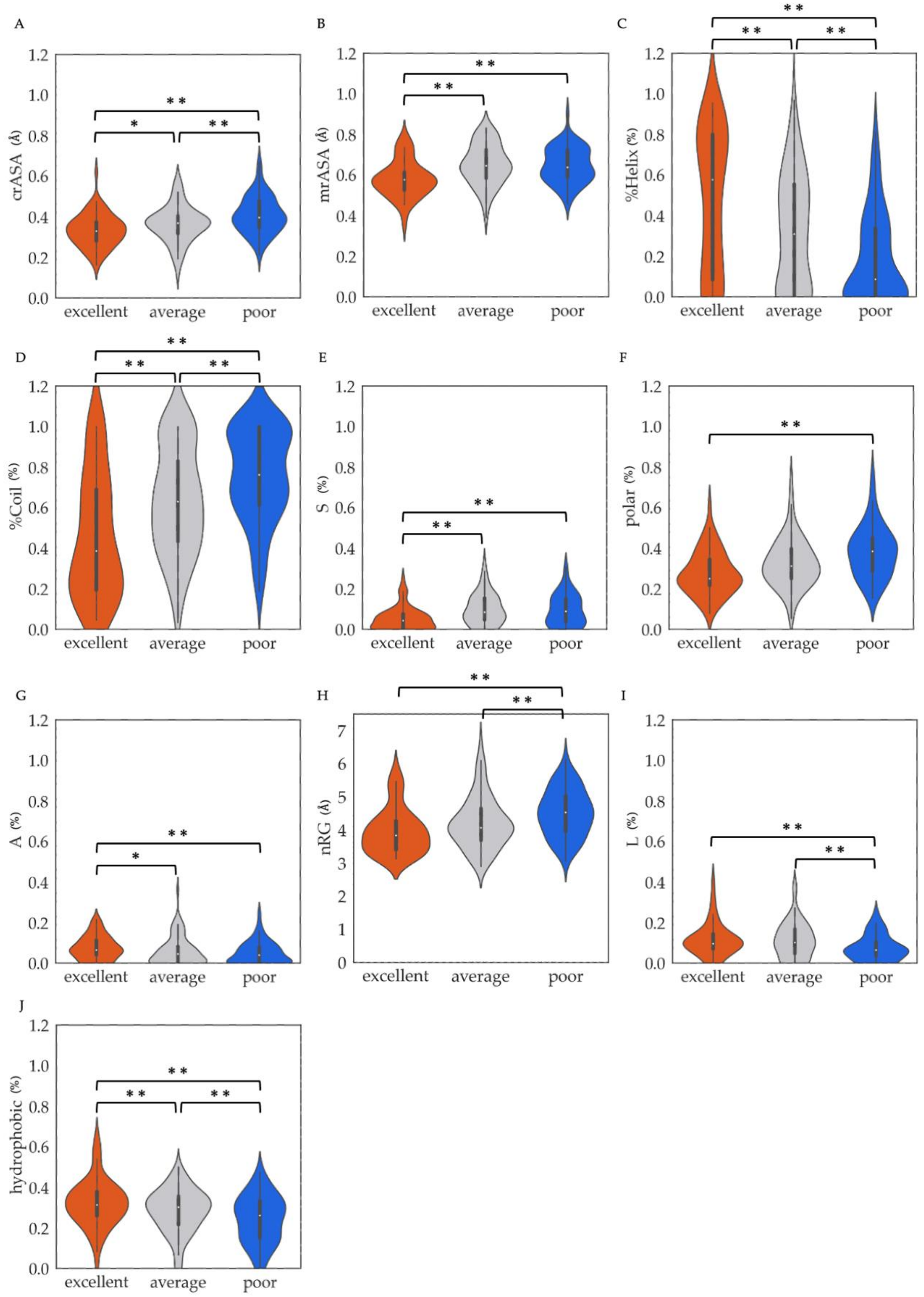

Figure S1: Distributions of structural and sequential features of ProS for excellent, average, and poor classes in violin plots. Features are denoted on the *y*-axis. Asterisk represents significant difference of the distribution with the Mann–Whitney *U*-test (one asterisk: 0.016 *p*-value, two: 0.010 *p*-value).

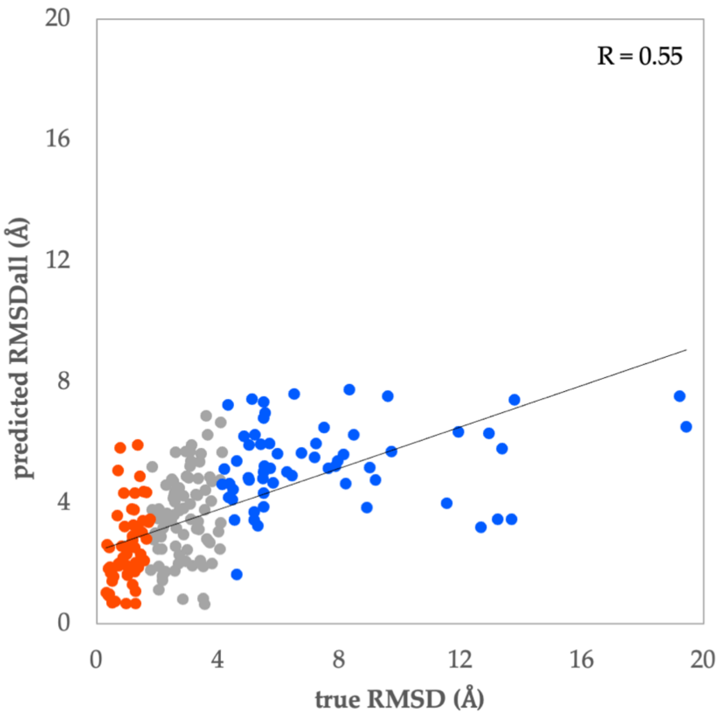

Figure S2: Multiple regression of RMSDs for all ProSs using 11 features. Blue, gray, and red dots represent ProSs in the excellent, average, and poor classes, respectively.

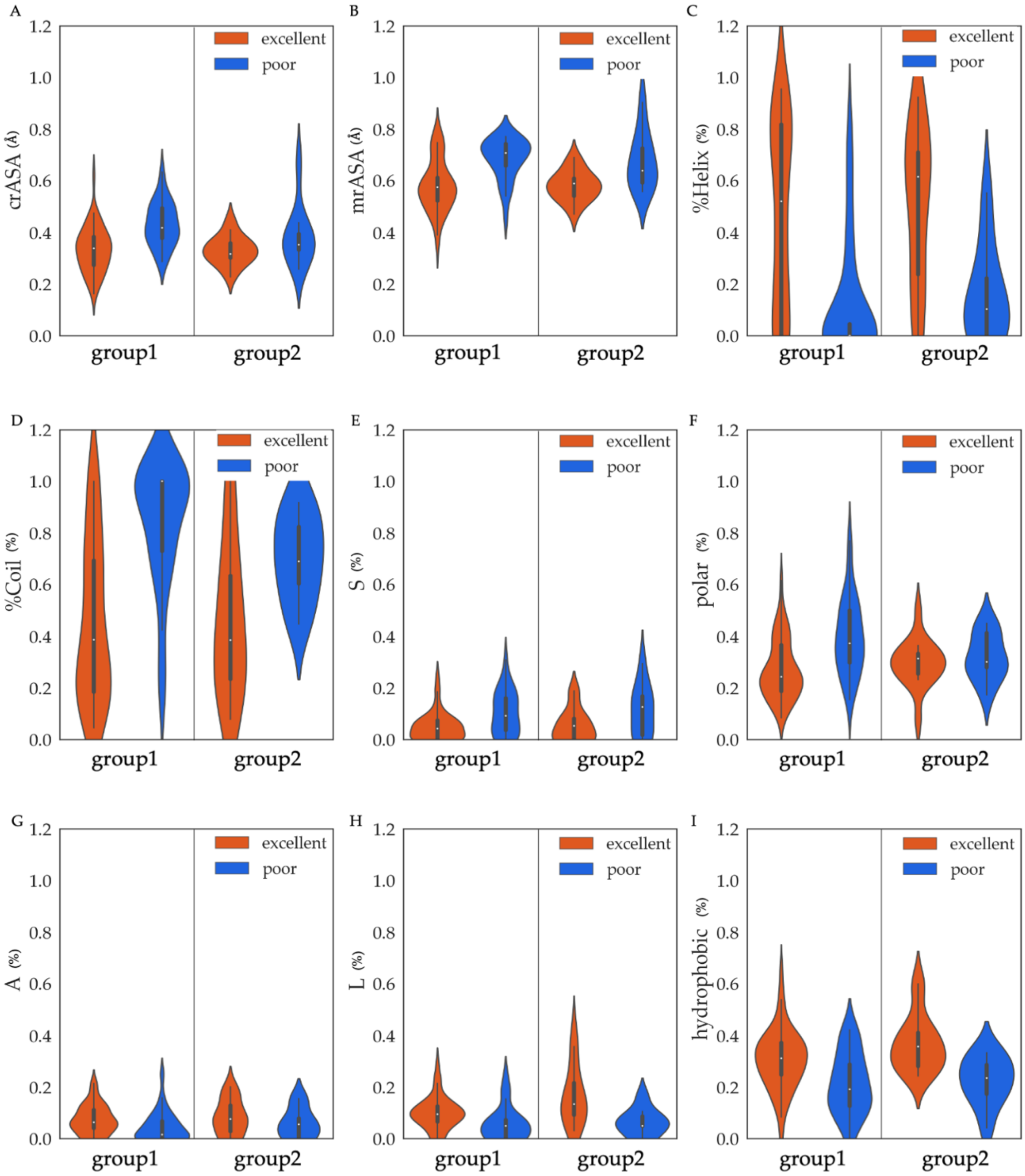

Figure S3: Distributions of structural and sequential features of group 1 and 2 ProSs in the excellent and poor classes. Features are denoted on the *y*-axis.

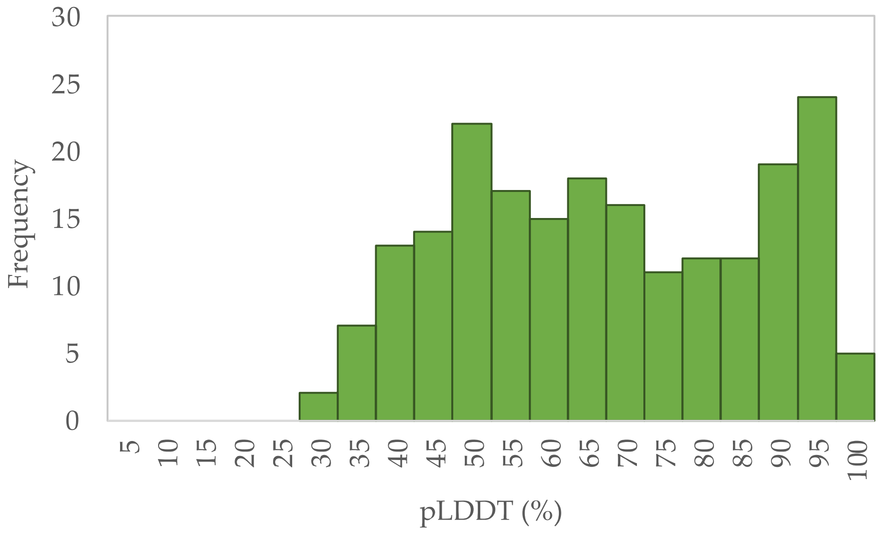

Figure S4: Distribution of pLDDT of non-redundant ProSs in IDEAL.
